# (State) empathy: how context matters

**DOI:** 10.3389/fpsyg.2025.1525517

**Published:** 2025-02-07

**Authors:** Katrin Heyers, Robin Schrödter, Lena Sophie Pfeifer, Sebastian Ocklenburg, Onur Güntürkün, Ursula Stockhorst

**Affiliations:** ^1^Biopsychology, Institute of Cognitive Neuroscience, Faculty of Psychology, Ruhr University Bochum, Bochum, Germany; ^2^Experimental Psychology II and Biological Psychology, Institute of Psychology, School of Human Sciences, Osnabrück University, Osnabrück, Germany; ^3^Institute of Exercise Science and Sport Informatics, German Sport University Cologne, Cologne, Germany; ^4^Cognitive Psychology, Institute of Cognitive Neuroscience, Faculty of Psychology, Ruhr University Bochum, Bochum, Germany; ^5^Department of Psychology, MSH Medical School Hamburg, Hamburg, Germany; ^6^Institute for Cognitive and Affective Neuroscience, MSH Medical School Hamburg, Hamburg, Germany

**Keywords:** empathy, increasing and decreasing factors, bottom-up process vs. top-down control mechanisms, trait, state

## Abstract

Empathy is a pivotal capacity that is essential for human interaction. It encompasses cognitive empathy, which is the ability to understand another individual’s emotional state, and affective empathy, which is to express an appropriate affective response to another person’s emotional state. Recent advancements in empathy research have highlighted the contextual nature of both cognitive and affective empathy, signifying their susceptibility to modulation by situational factors. Despite this progress, a comprehensive mechanistic understanding of empathy as a form of situated cognition that integrates both state and trait dimensions remains scarce. This review outlines the interplay of trait and state empathy and how state empathy emerges from a dynamic interplay between bottom-up processes and top-down control mechanisms. It further covers which situational factors increase versus decrease state empathy. In addition, to assist in selecting appropriate measurement tools for measuring trait and/or state empathy, the review categorizes existing empathy measurement instruments. Taken together, this review provides a roadmap for enhancing the efficacy of future empathy studies by: (1) outlining the current theoretical and methodological considerations for disentangling trait and state empathy; (2) organizing existing empathy measurement tools to aid researchers in selecting appropriate tools for future studies; (3) describing the interplay between bottom-up processes and top-down control mechanisms for state and trait empathy; and (4) reviewing factors that increase or decrease state empathy to prevent their potential interference and enable a more accurate assessment of empathy.

## (State) empathy: how context matters

According to a recent umbrella review “empathy is to understand, feel, and share what someone else feels with a clear self-other differentiation” (Håkansson Eklund and Summer Meranius, 2021, p. 306). This definition integrates two essential components, namely, how well an individual can perceive and understand the emotions of another individual (cognitive empathy), and the affective state an individual feels in response to others’ emotions (affective empathy). The long-term coexistence of multiple definitions may explain why a variety of measurement tools are available to assess empathy (Hall and Schwartz, 2019). Thus far, however, situational factors that modify empathy, thereby addressing the situatedness of empathy, have yet to be considered with regard to empathy for complex emotions. Following the work of Nezlek et al. (2001), Cuff et al. (2016) recently emphasized the importance of considering the interaction between trait capacities and state-related factors for individual empathic responses. Indeed, this may be crucial when establishing a comprehensive framework to explain the processes of empathy. The authors propose that an individual’s capacity for empathy consists of a stable trait component, akin to personality factors, and a state component influenced by specific situational factors like acute stress, pain, or mood (Cuff et al., 2016). Although the selection of different measurement tools for different aspects of empathy (e.g., self-report measures for trait empathy and task-based performance measures for state empathy) has become an implicit practice by researchers, standardized measures of empathy do not always integrate both aspects and sometimes even ignore this distinction. This results in ambiguity when interpreting whether differences in empathic responses reflect (a) trait capacities of an individual, or (b) state empathy influenced by the specific situation or natural condition in which the study was conducted. A comprehensive assessment of the various components of empathy that incorporates contextual factors that impact state empathy is necessary to address this issue. While studies have considered situational factors such as motivation for enhancing empathy (Weisz et al., 2021), a structured approach has yet to be taken to differentiate trait and state empathy.

The aim of the present review, therefore, is to comprehensively examine current theoretical and methodological considerations and empirical evidence for differentiating trait and state empathy. It comprises four main steps: (1) outlining the current theoretical and methodological considerations for disentangling trait and state empathy; (2) organizing existing empathy measurement tools to aid researchers in selecting appropriate tools for future studies; (3) describing the interplay between bottom-up processes and top-down control mechanisms for state and trait empathy; and (4) reviewing factors that increase or decrease state empathy to prevent their potential interference and enable a more accurate assessment of empathy.

## The theoretical differentiation between trait and state empathy

The Perception-Action Model (PAM; Preston and de Waal, 2002), one of the leading theoretical frameworks relating to empathy, is particularly relevant for establishing a theoretical definition of trait and state empathy. The PAM depicts a bottom-up process of empathy, meaning that initially both neural and cognitive representations of the emotional state of another person emerge within the individual. The framework was further developed to include top-down regulation processes that moderate the initial automatic component of empathy (de Vignemont and Singer, 2006; Preston et al., 2020), and that relate to executive functions, self-regulation mechanisms, or attention (Preston et al., 2020). Transferring this idea to trait versus state differentiation, we argue that the bottom-up process of empathy may be the result of the initial activation of trait empathy, while the top-down regulation may shape the final state empathy reaction. In general, trait factors are considered traits only when they demonstrate stability over time and consistency across contexts, state components are generally characterized by their variability in response to acute situations. They change over the lifespan and are influenced by contextual factors (Roberts and DelVecchio, 2000). In previous work conducted by van der Graaff et al. (2016), Zhao et al. (2021), and Zhao et al. (2022), trait empathy is considered the general ability to express empathy, while state empathy responses depend on the immediate context. In line with this hypothesis, Hall and Schwartz (2019) consider that empathy originates from a general capacity that is further influenced by situational or motivational factors, thereby emphasizing that the distinction between trait and state components of empathy has been proposed by various researchers.

On an experimental level, a small number of studies have recently approached empathy from a trait versus state perspective and investigated the extent to which the two concepts coincide (van der Graaff et al., 2016; Zhao et al., 2022; Zhao et al., 2021). For instance, in response to *sadness*, and thereby including the valence of the stimulus material, trait empathy [measured using the Interpersonal Reactivity Index (IRI) Davis, 1983] was positively associated with state empathy (measured by the rating of emotional film clips) both for cognitive and affective empathy measures (van der Graaff et al., 2016). The same pattern occurred in response to *happiness,* though less consistently (van der Graaff et al., 2016). Regarding the culture-sex interaction in empathy, both ethnic-group bias and sex-group favor (adapted according to the sex of the stimulus) were reported to vary for different groups (Caucasian versus Asian participants) in task-based state empathy measures (single-character portraits and documentary photos with emotional background) (Zhao et al., 2021). In their study, state empathy was measured using two sets of stimuli: single-character portraits and documentary photos with emotional background, with each set consisting of 24 pictures (depicting the following stimulus features 2 cultures, 2 sexes, and 6 emotions [happiness, anger, sadness, surprise, and fear, and neutral-peacefulness of the protagonists]). Data revealed significant two-way, three-way, and four-way interactions of the factors sex of the participant, and stimulus sex, as well as culture of the participant and stimulus culture, resulting in specific in-group vs. out-group relations. Furthermore, the effects partly varied depending on the specific emotion quality. The measurement tools to assess trait empathy, the IR and the Empathy Quotient (EQ, Baron-Cohen and Wheelwright, 2004) only allowed examining the effects of the participants´ culture (only found in females, with higher scores in Australian than Chinese women) and the participants´ sex (only found in Australians with higher scores in Australian women than Australian men). Future studies should consider the assessment of culture-sex interaction in both, state and trait measures of empathy.

This highlights the interaction of individuals’ trait characteristics (measured by self-report questionnaires) and state factors (Zhao et al., 2021). The trait versus state dissociation of empathy was further investigated from a neuroscientific perspective in a female sample: the resting-state activity patterns of the state empathy neural network consisting of the bilateral supplementary motor area and the middle cingulate cortex, as well as the left anterior insula and the inferior frontal gyrus, could predict trait empathy measures (Zhao et al., 2022). In essence, while the interplay between trait and state empathy is present, these nuanced distinctions underscore the need to continue investigating the relationship between the two dimensions.

Some studies have attempted to identify factors that influence empathy without differentiating between state and trait empathy. Cuff et al. (2016) compiled factors that vary within the individual, such as current cognitive load (Rameson et al., 2012), perceived power (Galinsky et al., 2006), perceived need to emphasize (Lishner et al., 2011), blame (Rudolph et al., 2004), mood (Pithers, 1999) or observer-target similarity (Håkansson Eklund et al., 2009) to the empathic response of the observer. Further factors that affect an observer’s expression of empathy are acute stress (Nitschke and Bartz, 2023; von Dawans et al., 2021; Wolf et al., 2015), attention (Zaki, 2014), emotions or affective states (Tamir, 2016; Thompson et al., 2019), motivation (Zaki, 2014) or the different hormonal phases in women’s menstrual cycles (Gamsakhurdashvili et al., 2021b). This will be discussed in more depth later in this review. Interestingly, other psychological constructs, for example, anxiety, have already been thoroughly investigated concerning the difference between trait and state, and distinct measurement tools have been developed (for an example, see the State–Trait Anxiety Inventory by Spielberger et al. (1971)). This indicates that such a distinction may increase our understanding of such psychological constructs. It is important to further view this differentiation from a developmental perspective: Jarvis et al. (2024) recently published a meta-analysis indicating that affective empathy may increase with age. Cognitive empathy seems to be relatively stable once individuals reach adulthood, only starting to decline after the age of 65 (Dorris et al., 2022). Yet, both affective and cognitive empathy seem to vary across the lifespan, thereby indicating that state measures of those concepts are susceptible to context-dependent changes. Although beyond the scope of this review, it should be acknowledged that investigating empathy, and more precisely trait and state empathy from a clinical perspective holds the potential to unravel nuances in trait and state empathy (for a comparison see Preston et al. (2020)).

Despite these research efforts, little is known about how current evidence relates to the distinction between trait and state empathy. Interestingly, research widely considers that empathy consists of two facets: cognitive versus affective empathy. This distinction, however, fails to explicitly acknowledge empathy as a situated capacity; nevertheless, the differentiation between cognitive and affective empathy may play a role in conceptualizing trait and state empathy. Situatedness or a situated capacity is the idea that contexts plays a pivotal role in expressing capacities and that a skill (or in the case of situated cognition a thought) is specific to a situation (Brown et al., 1989; Newen et al., 2018). We look at cognitive and affective empathy as both traits and states, and highlight the differences between empathy and its related constructs. We will review exemplary findings that suggest a distinction between cognitive and affective empathy from a trait versus state perspective. Of note, the factors reported to influence the different facets of empathy are exemplary as well. A comprehensive review of factors increasing and decreasing empathy will be provided at a later point.

### Cognitive and affective empathy as a trait and as a state

As mentioned above, definitions of empathy cover both cognitive and affective empathy (Cuff et al., 2016; Singer and Lamm, 2009). It has been shown that cognitive and affective empathy partly rely on different neural circuits (Shamay-Tsoory et al., 2009). This suggests that cognitive and affective empathy are independent to some extent. However, their specific interaction and interdependencies are still debated (Cuff et al., 2016; Preston et al., 2020).

It has recently been argued that affective empathy can occur without cognitive empathy but not vice versa because affective representations in the bodily state are necessary to understand the emotional state of another person (Preston et al., 2020). Notably, affective empathy is considered the result of an initial bottom-up process wherein the perception of external stimuli, e.g., the emotional state of another person, induces a representation in the observer itself (de Waal and Preston, 2017; Preston et al., 2020). On the contrary, cognitive empathy is thought to be a top-down process in which internal stimulation leads to cognitive empathy processes. Despite this difference in the origin of stimulation (external versus internal), and the sequence of processes (bottom-up to top-down process for affective empathy and top-down to bottom-up process for cognitive empathy), both affective and cognitive empathy activate affective representations in the observer, leading to similar representations in bodily states (de Waal and Preston, 2017; Preston et al., 2020).

The distinction between cognitive and affective empathy may also be crucial when discussing situational variables affecting state empathy. In this regard, twin studies estimating genetic and environmental portions suggest that affective empathy is more heritable than cognitive empathy, as demonstrated by a recent meta-analysis (Abramson et al., 2020). From this finding, one might conclude that affective empathy is more strongly determined by genetic factors than cognitive empathy, and in turn, that affective empathy may be more stable and cognitive empathy more situated. Cognitive empathy may thus be more susceptible to the potential effects of detrimental environmental factors, but also more responsive to interventions meant to increase empathy.

Preliminary findings contradict these conclusions. For example, Wolf et al. (2015) did not find changes in cognitive empathy measures after acute stress. In contrast, however, they report an increase in affective empathy following acute stress. Other studies report beneficial effects of acute stress on emotion recognition, a subcomponent of cognitive empathy (Domes and Zimmer, 2019). The apparent inconsistency in these results can be attributed in part to differences in the selection of measurement tools for empathy. For example, Wolf et al. (2015) implemented the Multifaceted Empathy Test in its Condensed and Revised Version (MET-core-2; Dziobek et al., 2011) to measure empathy, a task that includes the identification and the affective sharing of complex emotions. Alternatively, participants in a study by Domes and Zimmer (2019) were asked to decide whether one of two basic emotions or a non-emotional condition (angry, happy, or neutral) was present on pictorial stimuli. Such methodological disparities likely contribute to the mixed findings, an explanation that will be elaborated later in the manuscript.

The issue becomes even more complex when one considers that both cognitive and affective empathy lead to changes in bodily states (de Waal and Preston, 2017). Thus, the construct of embodiment must be recognized when discussing empathy. Embodiment is a psychological construct that refers to the idea that our thoughts, emotions, and behaviors are shaped by the physical body and its interactions with the environment (Newen et al., 2018). In the context of empathy, embodiment implies that bodily physical sensations influence the way we experience and respond to others’ emotions (Niedenthal, 2007). One implication of this approach is that perceiving another person’s emotions depends on the interaction between your bodily state and the emotions the other person displays (Niedenthal, 2007). This idea also implies that perceiving an emotion is always situated, and empathy should be considered an embodied process instead of a purely cognitive one.

In sum, because empathy is conceptualized as a complex and dynamic process influenced by a variety of internal and external factors, it is imperative to establish guidelines in measurement standards for empathy research. The recent theoretical and empirical developments in empathy research lead us to conclude that trait empathy represents an individual’s overall capacity for both cognitive and affective empathy. In contrast, state empathy is the specific expression of this capacity in a given situation. In this regard, the definition of Håkansson Eklund and Summer Meranius (2021) appears incomplete and requires expansion for clarity. We propose the following addition (*see italics*) to this definition: The *general* construct of *trait* empathy includes four factors: understanding, feeling, and sharing another person’s emotion with a clear self-other differentiation. *The expression of empathy is situated within both psychological and physiological factors that influence the empathic response.*

### Theoretical discrimination of trait and state empathy from related constructs

Past research on empathy has not only been challenged by methodological issues but also theoretical ones, specifically in differentiating empathy from its related constructs (Cuff et al., 2016). A central factor in discriminating empathy from other constructs is self-other differentiation (de Vignemont and Singer, 2006). In (both trait and state) empathy, the observer is aware that the affective state of interest originates in another person and not in oneself. In contrast, when experiencing emotional contagion for example, the observer acknowledges an affective state but is unable to determine where it originated. Thus, the observer believes that the observed affective state comes from themself and fails to differentiate between sources of origin (Cuff et al., 2016).

It is also important to disentangle state empathy from pro-social behavior. Pro-social behavior is broadly defined as any action or behavior that promotes welfare in others (Pfattheicher et al., 2022). State empathy is the situated expression of empathy that can, but does not have to, be expressed in behavior. The question then arises of how state empathy and prosocial behavior are connected.

It has been suggested that (state) empathy constitutes the basis for showing prosocial behavior (Stevens and Taber, 2021). However, empathy and prosocial behavior do not seem to act on each other directly. Instead, it has been proposed that empathy resulting in prosocial behavior is mediated by compassion (Stevens and Taber, 2021). Compassion is defined as the ability to understand when another person is suffering and feeling emotionally connected to that person (Strauss et al., 2016). Furthermore, the observer can understand the common ground of this emotion, tolerate the (potentially negative) emotions that result in themself, and finally act or develop the motivation to act (Strauss et al., 2016). Stevens and Taber (2021) postulate that while compassion leads to prosocial behavior, empathy does not do this automatically. Regardless, empathy is considered an essential component of the emergence of compassionate feelings and thus prosocial behavior (Lim and DeSteno, 2016).

Two more distinctions are important to note. First and foremost, the difference between emotion recognition and empathy plays a central role in empathy research. Emotion recognition is a cognitive ability that enables the recognition of basic and complex emotions in others (Domes and Zimmer, 2019; Gamsakhurdashvili et al., 2021a). It is generally considered to be one facet of cognitive empathy (Cuff et al., 2016; von Dawans et al., 2021). Beyond this, cognitive empathy includes further processes such as mentalizing (von Dawans et al., 2021). Basic emotions typically cover happiness, sadness, fear, anger, surprise, and disgust, and are recognized across cultures (Fridenson-Hayo et al., 2016). In contrast, complex emotions are culturally dependent and rely on the context in which they occur (Fridenson-Hayo et al., 2016). Second, for complex emotions, it is worthwhile making a distinction between empathy for pain vs. empathy for other complex emotions. Empathy for pain has been researched extensively in behavioral paradigms (Gonzalez-Liencres et al., 2016; Lamm et al., 2007; Tomova et al., 2017), but only a few studies consider state empathy measures that target more complex, situated emotions as assessed by the MET-core-2 (e.g., highly satisfied, relaxed, or jubilant as positive emotions, and terrified, frustrated or desperately unhappy as negative emotions) while measuring both, cognitive and affective empathy (Drimalla et al., 2019; Dziobek et al., 2011; Dziobek et al., 2008; Gamsakhurdashvili et al., 2021a; Gamsakhurdashvili et al., 2021b; Wolf et al., 2015). Findings from previous studies targeting empathy for pain have been generalized to the wider construct of empathy (Lamm et al., 2007; Tomova et al., 2017). However, Timmers et al. (2018) showed that there are unique neural correlates of empathy for pain and that because a distinction between empathy for pain and empathy for other emotions exists, any generalizations should be made with caution (Timmers et al., 2018). These different components reveal that empathy is a nuanced concept and that measurement tools must be adjusted to the specific aspect of the research question. To the extent that it is possible, this review focuses on measurements of empathy for complex emotions.

## The methodological differentiation between trait and state empathy

Previous studies have tried to measure empathy via self-report measures, task-based performance / behavioral measures, or neuroimaging. However, because studies have yet to systematically match the theoretical definition of empathy to the measurement tool used to assess empathy or an empathy-related construct, Hall and Schwartz (2019) call for an adapted multitrait-multi-method approach to assess empathy in order to increase homogeneity and “accommodate both empathic traits and empathic states” (Hall and Schwartz, 2019, p. 235). This review considers the extent to which the distinction between trait and state empathy is reflected in methodological approaches used thus far. The following chapter outlines how the most prominent tools for measuring the empathy construct currently address the distinction.

### Self-report measures

In self-report measures, participants are asked to evaluate their empathic abilities or situational expression by choosing a response based on how much they agree or disagree with an item (e.g., “I really get involved with the feelings of the characters in a novel.”; Davis, 1983). Hall and Schwartz (2019) have recently gathered an overview of the most frequently used self-report empathy measurement instruments. Similarly, Yu and Kirk (2009) have listed available measurement tools. Based on these two reviews, we have compiled an overview of self-report measures of empathy in Table 1. Note that to refrain from a subjective bias when selecting the list of measurement tools included in this review, we decided to base our list solely on the systematic review by Yu and Kirk (2009) and the quantitative review by Hall and Schwartz (2019). Thereby, the list is not exhaustive but aims to summarize the most common approaches. It is important to acknowledge, that by using this approach to select measurement tools, some tools that are also used in research are not included in this review [e.g., the Emotional Contagion Scale by Doherty (1997) and the Emotional Empathy Scale by Mehrabian and Epstein (1972)]. To facilitate the choice of an appropriate measurement tool, it is essential to know which tools address which facets of empathy (cognitive versus affective versus both), and if they distinguish between trait and state empathy.

**Table 1:** Self-report measures assessing empathy.

Self-report measure	Reference
Barrett-Lennard Relationship Inventory (BLRI)^++^	Barrett-Lennard (1962)
Balanced Emotional Empathy Scale (BEES)*	Mehrabian (1996, 1997)
Basic Empathy Scale (BES)^++^	Jolliffe and Farrington (2006)
Batson Scale	Batson et al. (1987)
Carkhuff Indices of Discrimination & Communication (CIDC)^++^	Carkhuff (1969)
Child Victim Empathy Distortions Scale (CVEDS)^++^	Beckett and Fisher (1994)
Consultation and Relationship Empathy (CARE)^++^	Mercer et al. (2004)
Emotional Intelligence Scale (EIS)	Schutte et al. (1998)
Empathy Construct Rating Scale (ECRS)^++^	La Monica (1981)
Empathy Quotient (EQ)	Baron-Cohen and Wheelwright (2004)
Hogan Empathy Scale (HES)	Hogan (1969)
Interpersonal Reactivity Index (IRI)	Davis (1983)
Jefferson Scale of Physician Empathy (JSPE)^++^	Hojat et al. (2001)
Layton Empathy Test (LET)^++^	Layton (1979)
Perception of Empathy Inventory (PEI)*	Wheeler (1990)
Questionnaire of Cognitive and Affective Empathy (QCAE)	Reniers et al. (2011)
Questionnaire Measure of Emotional Empathy (QMEE)	Mehrabian and Epstein (1972)
Rape Empathy Scale	Deitz and Byrnes (1982)
Reynolds Empathy Scale^++^	Reynolds (2000)
Scale of Ethnocultural Empathy (SEE)	Wang et al. (2003)
Socio-emotional Questionnaire (SEQ)^++^	Bramham et al. (2009)
Toronto Empathy Questionnaire (TEQ)	Spreng et al. (2009)

List in alphabetical order, based on Hall and Schwartz (2019) and Yu and Kirk (2009). The asterisks indicate: *The scale was originally listed in Hall and Schwartz (2019) or Yu and Kirk (2009) but the original manuscript could not be retrieved; therefore, the scale was excluded from further categorization and discussion in this review. ^++^ This scale only assesses aspects of empathy in specific but not the general population.

In addition, Vieten et al. (2024) recently published a very concise and comprehensive overview of both, empathy and compassion measurement tools. While these authors have focused on including both constructs, the present review solely focuses on the work by Hall and Schwartz (2019) and Yu and Kirk (2009) and thereby on empathy measurement tools and the distinction of trait and state empathy.

### Task-based performance and behavioral measures

**Figure 1:**
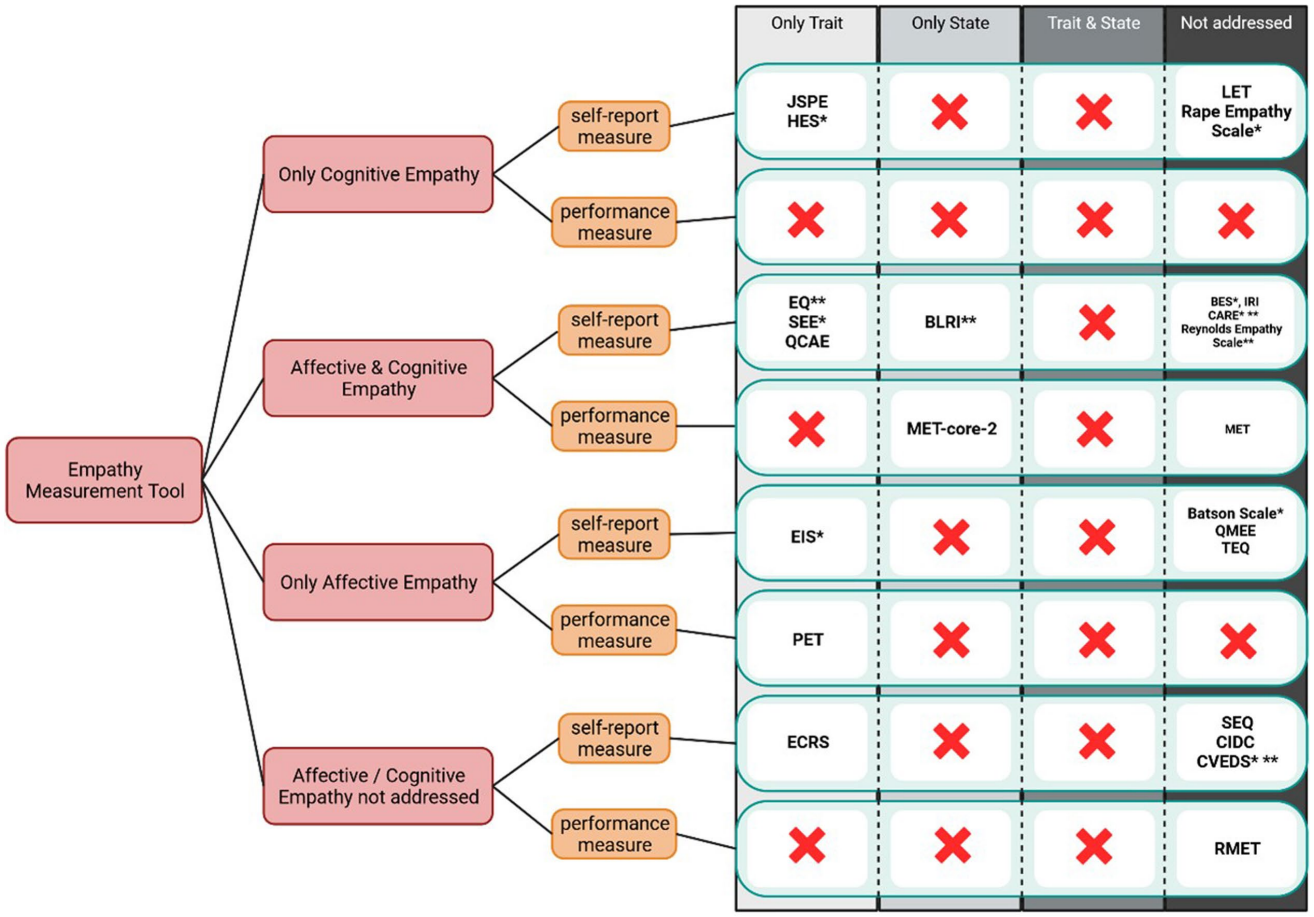
Diagram to select the appropriate Empathy Measurement Tool. This diagram sorts current empathy measurement tools by three factors: (1) whether the original manuscript addresses either cognitive or affective empathy separately or combined or if it does not address these components (pink boxes); (2) whether it is a self-report or a performance measure (yellow boxes) and (3) whether the original manuscript addresses if the tool measures empathy as a trait, as a state, or both trait and state combined or if it does not address it (green boxes). A red cross indicates that, to the best of our knowledge, there is no current empathy measurement tool available for this category. Please note that the categorization of the Balanced Emotional Empathy Scale (BEES; Mehrabian, 1996) and the Perception of Empathy Inventory (PET; Wheeler, 1990) are not included in this figure despite their original listing in Yu and Kirk (2009) or Hall and Schwartz (2019) due to the lacking availability of the original manuscript. The asterisks indicate: *Category (either cognitive/affective or trait/state) was not directly addressed in the manuscript but could indirectly be concluded. **Cognitive and Affective Empathy was addressed but is not being measured separately. BLRI, Barrett-Lennard Relationship Inventory; BES, Basic Empathy Scale; CIDC, Carkhuff Indices of Discrimination & Communication; CVEDS, Child Victim Empathy Distortions Scale; CARE, Consultation and Relationship Empathy; EIS, Emotional Intelligence Scale; ECRS, Empathy Construct Rating Scale; EQ, Empathy Quotient; HES, Hogan Empathy Scale; IRI, Interpersonal Reactivity Index; JSPE, Jefferson Scale of Physician Empathy; LET, Layton Empathy Test; MET, Multifaceted Empathy Test; MET-core-2, Multifaceted Empathy Test Condensed and Revised Version; PET, Pictorial Empathy Test; QCAE, Questionnaire Measure of Emotional Empathy; QMEE, Questionnaire Measure of Emotional Empathy; SEE, Scale of Ethnocultural Empathy; SEQ, Socio-emotional Questionnaire; TEQ, Toronto Empathy Questionnaire; RMET, Reading the Mind in the Eyes Test. Created with Biorender.com.

Performance measures, in contrast to self-report measures, require participants to make a (behavioral) forced choice between different alternatives with one of the alternatives being the correctly identified emotion. This opens up the possibility of measuring empathy based on responses as participants do not subjectively have to rate their empathic skills. In the Reading the Mind in the Eyes Test, participants are instructed to identify the emotional state of the protagonist by evaluating the expression of a pair of eyes on a picture and choosing one of four alternatives (RMET; Baron-Cohen et al., 2001). Other frequently used tests are the Pictorial Empathy Test (PET; Lindeman et al., 2016) and the Multifaceted Empathy Test, both in its original version (MET; Dziobek et al., 2008) and a Condensed and Revised Version (MET-core-2; Drimalla et al., 2019; Dziobek et al., 2011). In line with the questions raised in the previous chapter on self-report measures, we provide a systematic categorization of empathy measurements. The tools are (1) categorized based on whether they assess cognitive empathy, affective empathy, both constructs, or neither, (2) further classified as a self-report measure or performance measure, and (3) grouped on whether they specifically address trait empathy, state empathy, both constructs, or neither. In addition to the tools addressed by Yu and Kirk (2009) and Hall and Schwartz (2019), we also included the MET (Dziobek et al., 2008), the MET-core-2 (Drimalla et al., 2019; Dziobek et al., 2011), the BES (Jolliffe and Farrington, 2006), the PET (Lindeman et al., 2016) and the RMET (Baron-Cohen et al., 2001) in Figure 1 based on the frequency of their use in research. Where possible, we have included a citation of the sentence upon which we based our decision in Appendix Table A. We based the categorization not on how these measures have been used in the past, but solely on what is stated in their respective manuals. This caused certain challenges; for example, we categorized the RMET (Baron-Cohen et al., 2001) as “not addressed” even though it has been used as a state measure in most studies. Similarly, the IRI (Davis, 1983) is frequently used as a trait measure of empathy but the original manuscript did not state specifically if this tool is used to measure trait or state empathy. As we believe this to be the most objective approach, we ask that researchers address the distinction between trait and state empathy in their manuals. Lastly, we want to point out that some information displayed in the table was adapted and extended based on the work by Yu and Kirk (2009).

As depicted in Figure 1, a high number of self-report measures cover trait empathy. In contrast, to our knowledge, only one self-report measure has been developed to approach state empathy, namely the BLRI (Barrett-Lennard, 1962), and no self-report measure specifically targets both, trait and state empathy in one tool. While Batson et al. (1987) mentioned in their manual of the Batson scale that it is advisable to establish a clear conceptual distinction between variations in empathic emotion experienced in specific situations, and that the construct gauged by self-report indicates a more general concept, the authors did not include two separate measures for trait and state empathy.

Figure 1 outlines that most measurement tools fail to define whether the self-report measure targets trait or state empathy. Although this can be indirectly inferred from the wording used in some of the items of the questionnaires, we want to emphasize that the authors should address the issue specifically to evolve and further define the correct use of the respective measurement tool. Correspondingly, performance measures were classified as a trait measurement tool, a state measurement tool, or did not address the distinction at all. We consider performance measures an appropriate means for assessing situated empathy that is influenced by contextual factors, e.g., the testing environment. The extent to which a performance measure can assess trait empathy via a repeated-measure design deserves further investigation.

One of the few studies to have addressed the difference between trait and state empathy was conducted by Zhao et al. (2021). They used both the IRI (Davis, 1983) and the EQ (Baron-Cohen and Wheelwright, 2004) as measures for trait empathy, and an adapted version of a task-based empathy measure by Neumann et al. (2013) to assess state empathy. Similarly, van der Graaff et al. (2016) measured trait empathy using the IRI (Davis, 1983) and state empathy using an adapted task-based empathy assessment that included watching emotionally loaded film clips, a subjective rating, and identification of the emotion. The selected videos for the state empathy task included four different clips representing either happiness or sadness and were taken from Dutch documentary films (van der Graaff et al., 2016). In addition, prior to the task as well as in between emotional video clips, participants watched fragments of an aquatic video fostering relaxation (van der Graaff et al., 2016). Notably, both studies used a self-report measure for trait empathy and a performance measure for state empathy; a division that we favor as well.

Given the classification, tools such as the MET (Dziobek et al., 2008) or the RMET (Baron-Cohen et al., 2001) have further limitations. For example, the extent to which existing performance measures of empathy can distinguish simple emotion recognition from cognitive empathy has been questioned (Preston et al., 2020). Both the RMET and the MET ask participants to recognize the emotional state of a target on pictorial stimuli but fail to include higher-level processing steps (Preston et al., 2020).

At this point, it is important to note that in addition to self-report and performance measures, neural activity might serve as a further category to assess empathy. To the best of our knowledge, only one study has specifically targeted the distinction between trait and/or state empathy from a neuroimaging perspective. Zhao et al. (2022) identified a neural network representing state empathy that included the bilateral middle cingulate cortex, the bilateral supplementary motor area, the left inferior frontal gyrus, and the anterior insula. The authors could link intrinsic brain activity in these regions to trait empathy measures conducted using the IRI (Davis, 1983). Yet, as far as we know, no neural network representing trait empathy distinctively from state empathy has been identified. We decided not to include neuroimaging measures as a third category in this review as studies approaching empathy from this perspective usually combine their measures with a self-report and/or a task-based approach to measure empathy.

It is important to apply caution if considering most measures as indicators of trait empathy. Performance and self-report results may be subject to the influence of situational or contextual factors during data collection. Therefore, it is crucial to interpret such measurements with care and account for potential sources of variability. In sum, Figure 1 shows that indications, of whether existing tools measure trait and/or state empathy are lacking, a problem that may partially explain inconsistencies in past empathy research and that can easily be addressed. Our above categorization enables researchers to select appropriate tools for their research questions and study designs, and facilitates the comparison of findings across studies.

In addition to differentiating between trait and state empathy, we further summarized whether respective measurement tools address the cognitive and/or affective domains of empathy. Older empathy measurement instruments in particular tended not to address this difference. This might be due in part to more recent theoretical developments since the differentiation between cognitive and affective empathy may have been established after the development of older tools.

Taken together, the question remains whether or not, and to what extent, existing measurement tools should be adapted. We argue that separating trait versus state empathy does not necessarily lead to new measurement tools. Instead, two advances should be made: (1) authors should clearly state if their experiment targets trait and/or state empathy, choose the appropriate tool, and argue why the tool is suited for the trait and state dimension; and (2) when performing empathy experiments, authors should gather information on situated influences that potentially modulate state empathy and include them as co-variances in the analyses. For this to happen, a consensus needs to be reached on which individual factors modify state empathy. This topic will be discussed in the next chapter.

## Factors to consider when measuring state empathy

### The situated framework to approach empathy as a state

As discussed earlier, current theoretical approaches consider empathy to be a capacity with expressions that are situated within and influenced by contextual factors (Hall and Schwartz, 2019). Both affective and cognitive empathy are assumed to include a bottom-up process as well as a top-down control mechanism (de Waal and Preston, 2017; Hall and Schwartz, 2019; Preston and de Waal, 2002; Singer and Lamm, 2009). Empathy for pain is the main area considered in terms of neural correlates of empathy. In this context, studies show that neural networks related to empathy processes (mainly insula and anterior cingulate cortex, ACC) are activated even when participants are not explicitly asked to emphasize. This is understood as the initial bottom-up process that cannot be controlled by individuals (Singer and Lamm, 2009). Only later can the empathy process be regulated by top-down control mechanisms. Although a clear distinction between bottom-up and top-down processes is challenging to determine empirically, a theoretical distinction between the two holds value by providing testable predictions and hypotheses for future studies. Furthermore, several studies illustrate that the empathic reaction after stimulus onset alters over time, indicating that there are fast, intuitive, and slow deliberative processes influencing the final expression of empathy (for an overview of examples for both processes in more detail see Singer and Lamm, 2009).

**Figure 2:**
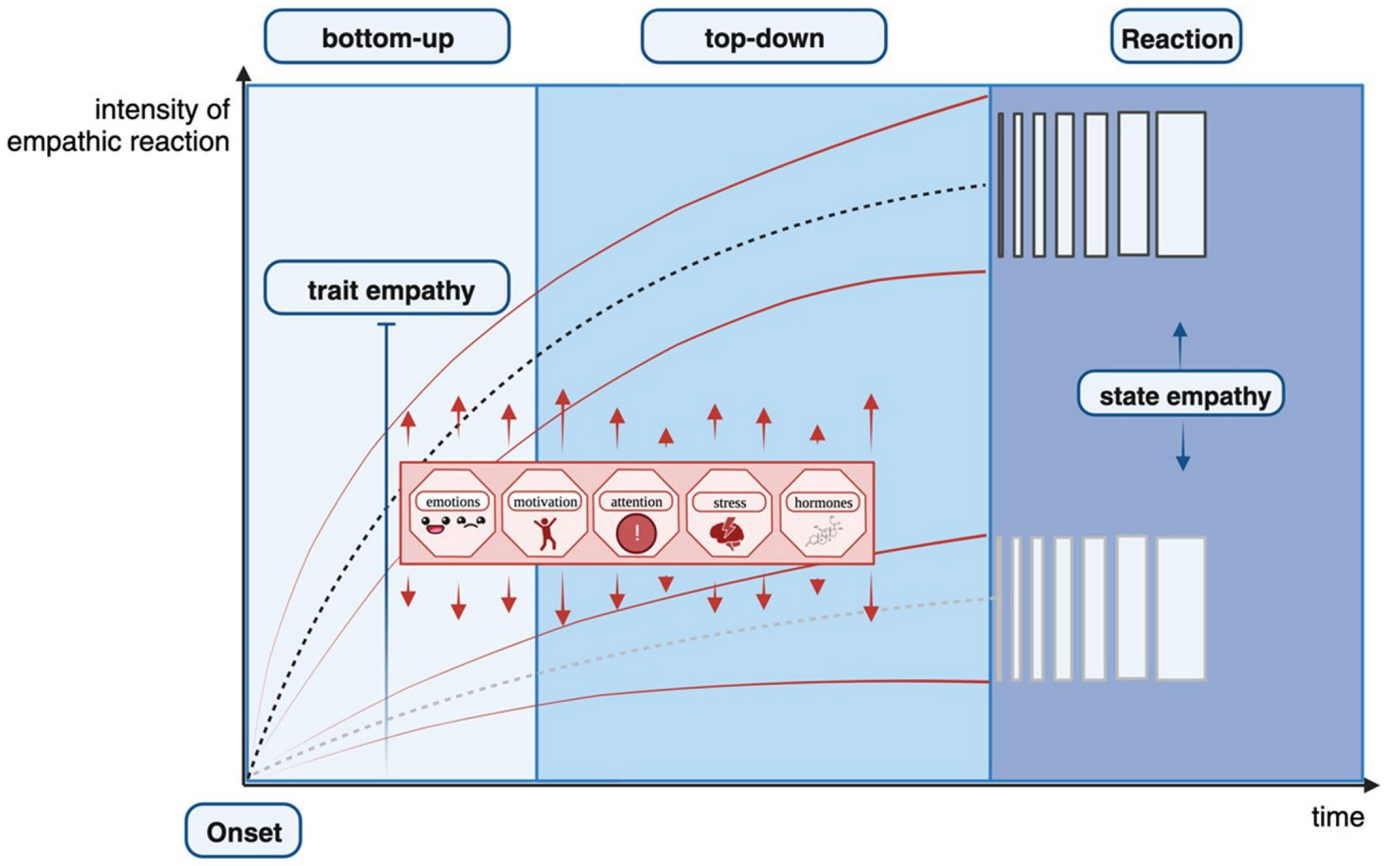
Situated framework for trait versus state empathy. The figure is a graphic description of the temporal sequence of the bottom-up process and the top-down control process. Further incorporated is the intensity of the empathic reaction as well as the trait versus state component. The *x*-axis represents the temporal domain starting with the onset of an empathic reaction. On the *y*-axis, the intensity of the empathic reaction is displayed. During the first process, the bottom-up process, trait empathy determines the intensity of the empathic reaction as initiated by the different dotted lines. Starting during the bottom-up process and extending to the second process of the empathic reaction, namely the top-down control process, situational factors influence the steepness of the initial empathic reaction and have the potential to both increase and decrease the empathic reaction, hereby indicated through the red arrows and red lines. During the third process, the empathic reaction is displayed, which is considered state empathy as it resulted from the process described above. Created with Biorender.com.

Empirical studies on empathy for more complex emotions are scarce. Despite this, it can be expected that complex emotional mechanisms work similarly. Building on the approach taken by Thompson et al. (2019), we conceptualize empathy as a process by adding the trait versus state perspective. We propose the following idea: when an empathic reaction is initiated (for example by an external stimulus like a crying family member), the trait empathy measure equals the general ability and functions as a stable multiplicator that determines the magnitude (steepness) of the initial, intuitive bottom-up process of the empathic reaction. In addition to trait empathy, various situational factors influence the intensity of the initial, intuitive bottom-up empathic reaction. For example, Morel et al. (2012) report contextual influences on face perception as early as 60 ms. Considering the short time frame, this influence can be attributed to the bottom-up process. Concerning top-down control mechanisms, different emotion regulation strategies reportedly either increase or decrease state empathy (Jauniaux et al., 2020; Thompson et al., 2019). Furthermore, similar to the bottom-up process, contextual factors can influence the top-down process; these situational factors may lead either to an increase or decrease in the initiated intensity of an empathic reaction. Consequently, the result of this process is the situated expression of trait empathy, measured in most studies investigating empathy (state empathy). We consider this approach to work equally for both cognitive and affective empathy processes. For a visualization, see Figure 2.

The presented idea implies that there is an inherent difference between measuring the empathic reaction shortly after the stimulus onset or after a longer time. The first measure reflects mainly the bottom-up process consisting of the general trait capacity while the latter represents the outcome of both the bottom-up process and the top-down regulation. Thus, the crucial variable for measuring task-based empathy is the time frame for indicating an individual’s empathic response. In a recent study, a 5 s time pressure was identified as a valid method to induce intuitive thinking in decision-making paradigms (Isler and Yilmaz, 2023). Although response time is usually recorded in empathy studies, it is rarely interpreted, and its impact is generally underestimated. Measurement standards for short-term empathic measures (for example <5 s) and long-term empathic measures (for example >10 s) are needed; for a comparison see Isler and Yilmaz (2023). Both approaches are important for understanding the different aspects of empathy. However, researchers must decide beforehand which aspect they intend to measure and design their experiment accordingly. We believe that the lack of time control contributes to the large variance between results in studies measuring empathy, and that mitigating this shortcoming could be a simple and cost-efficient way to improve empathy research.

The presented theoretical framework and its implications are in line with the following results: in two studies, individuals with self-reported high trait empathy were compared to individuals with low trait empathy as measured with the EQ (Baron-Cohen and Wheelwright, 2004; Rameson et al., 2012). Participants were instructed to either look at photos and empathize with the people in the photo, or look at photos while remembering an eight-digit number (passive condition but with a high cognitive load). Remarkably, participants with high levels of trait empathy exhibited heightened expressions of empathy under cognitive load compared to participants characterized by lower trait empathy (Rameson et al., 2012). This suggests that under conditions of high cognitive load, individuals with high-trait empathy report higher intensities of empathic reactions than individuals with low-trait empathy. Considering our approach, the steepness of the level of intensity that reflects trait empathy determines the level of intensity of the state empathy reaction. Although the assumption regarding time control remains untested, its validity could be determined if future studies implement two groups in task-based empathy measures like the MET-core-2. The first group would be forced to respond within 5 s of stimulus onset while the second group could not respond until 10 s after stimulus onset. This would ensure that the empathetic reaction of the first group was primarily based on the bottom-up process while the second group’s response was based on a mixture of the bottom-up and top-down processes. If the responses between groups varied significantly, the results would suggest that the distinction between the two processes is valuable and should be made in future research.

It is critical to note that trait and state empathy may not be entirely independent of one another, contrary to what might be inferred from the model presented above. Determining the relationship between trait and state processes—whether they are interdependent or distinct—requires experimental intervention studies. Crucially, such studies must employ comprehensive measurement tools, as previously emphasized, that assess both trait and state components of empathy.

Finally, it is important to have a basic understanding of the factors that increase or decrease the empathetic response when planning an experiment to reduce the risk of opposing factors falsely attenuating the effects of one another. Thus, the following chapters briefly outline increasing and decreasing factors that have been identified in previous studies.

### Increasing factors

This chapter examines internal/intraindividual and external situational factors that govern the presence of state empathy and serve as increasing factors.

#### Motivation

Motivation can be understood as a cognitive process that initiates purposeful goal-directed behavior (Wasserman and Wasserman, 2020). It is thought to influence the interpretation and appraisal of a given situation, thereby facilitating the emergence of an empathic response (Nitschke and Bartz, 2023; Preston et al., 2020).

However, it is still debated how exactly motivation shapes empathy. The literature differentiates between intrinsic and extrinsic motivation. Intrinsic motivation is generally understood as a form of motivation where a person does an activity simply because of the activity itself (Ryan and Deci, 2020); it thus arises in the person and is not based on the environment (Hendijani et al., 2016). Transferring this to empathy means that someone is empathic simply because they find intrinsic pleasure in the empathic action. According to the self-determination theory by Ryan and Deci (2000), autonomy is a critical driving factor increasing intrinsic motivation. Strikingly, this effect has been replicated and established in different contexts (Ryan and Deci, 2020). Empathy has been shown to encourage more helping behavior the more autonomous motivation occurs (Pavey et al., 2012). Motivation is currently regarded as the bridge between empathy and prosocial behavior. However, research is scarce on experimental approaches that connect autonomous motivation and the occurrence of empathy. Future research should aim to investigate the extent to which the self-determination theory parameter also applies to the occurrence of empathy.

Extrinsic motivation comes from external factors such as rewards, recognition, or pressure from others (Hendijani et al., 2016). The expectation of reward (Hendijani et al., 2016) can be considered an extrinsic motivation based on the situation or environment. Only a few studies have addressed the influence of reward on different aspects of empathy. Sims et al. (2012) showed that conditioned reward associated with different faces influenced the level of facial mimicry expressed, with higher reward inducing higher levels of facial mimicry. Facial mimicry is a subcomponent of empathy and consists of the facial mimic expression of emotions, or at least the emotional valence of an empathized emotion, that matches the emotion expressed by the counterpart (Drimalla et al., 2019). Haffey et al. (2013) not only found a similar effect of reward on mimicry, but further showed that trait empathy, measured using the EQ (Baron-Cohen and Wheelwright, 2004), predicted the level of automatic mimicry. In line with this, the mirror neuron system is reportedly influenced by reward (Trilla Gros et al., 2015). Notably, these studies used social rewards (e.g., faces or hands of human individuals) and mimicry as subcomponents of empathy or as markers of activity in the mirror neuron system. Taken together, it would be valuable to examine whether these effects are restricted to the subcomponents of empathy or apply to the broader concepts of cognitive and affective empathy as well.

#### Motives and emotion regulation strategies

Zaki (2014) identified six key motives (three avoidance-related, three approach-related) that modify the empathic outcome: (1) avoiding pain, (2) avoiding costs, (3) avoiding interferences, (4) approaching capitalizations, (5) approaching affiliation, and (6) approaching desirability. Such motives are intertwined with different emotion regulation strategies that allow for coping with a situation. An emotion regulation strategy is a method to modulate one’s emotional state to ensure optimal functioning in an environment and to uphold and improve well-being (Stevens and Taber, 2021). Emotion regulation strategies have recently been proposed as both increasing and decreasing factors for state empathy, depending not only on the specific strategy but also on the valence of the emotion (Jauniaux et al., 2020). Investigating complex emotions using an emotion regulation strategy that up-regulates the intensity of one’s emotional state (e.g., by taking the first-person perspective in a cognitive reappraisal process) led to higher state empathy, compared to a method that down-regulates one’s emotional state. In addition, state empathy was higher for negative social stimuli compared to positive social stimuli (Jauniaux et al., 2020). Weisz and Zaki (2017) emphasize that identifying the core motives driving empathic behavior can aid in designing effective empathy training programs, thereby enhancing the development and efficacy of such interventions. We take their approach a step further by suggesting that incorporating motive assessment during empathy experiments could offer valuable explanations for mixed findings in previous studies. This consideration can contribute to a more comprehensive understanding of empathy-related research outcomes.

#### Mindfulness and focus of attention

Attention is a key concept in psychology, involving selectively focusing on specific aspects over others (Posner and Petersen, 1990). Mindfulness is defined as non-judgmental awareness (Donald et al., 2019) and is considered one method for guiding one’s attention to the given moment and to the thoughts and emotions of an ongoing situation. As such, mindfulness has been explored for its potential to enhance state empathy (Donald et al., 2019). Recent research suggests a connection between mindfulness and empathy although formal mindfulness training has not consistently resulted in increased empathy (Cooper et al., 2020). Authors of one meta-analysis point out that methodological difficulties must be considered when interpreting their finding that meditation can boost empathy, and that future approaches should clarify any inconsistencies before building upon these results (Kreplin et al., 2018). Although prosocial behavior and empathy are distinct, it is notable that Luberto et al. (2018) identified a positive impact of meditation on prosocial behavior. It would be interesting to investigate the extent to which this impact could be expanded to empathy.

#### Emotions and affective states of the observer

Emotions are central in social interactions and are one of the most prominent topics in psychological research (Tamir et al., 2016). Emotions encompass physiological components, appraisals, expressions, and behaviors that shape an individual’s relationship with their environment (Tamir et al., 2016). Understanding how emotions influence state empathy is challenging due to the versatility of the expression of emotions and their subjective perception. One affective state discussed in the context of empathy is compassion. Personal distress, a potentially decreasing factor of empathic reaction (to be discussed later in the manuscript) (Kim and Han, 2018), may be counteracted by compassion. Whereas empathy is conceptualized as a self-directed emotion, compassion is considered an other-related emotion and leads to positive feelings such as love (Lantos et al., 2023). Interestingly, the potential for compassion training to increase positive affect (Klimecki et al., 2014), and in turn state empathy, has recently been acknowledged. More precisely, compassion training led to a decrease in activity in the respective neural regions connected to empathy for pain (Klimecki et al., 2014). In addition, a recent study used loving-kindness meditation training as a form of compassion-based training to increase self-report empathy measures, namely the JSPE (Hojat et al., 2001). As part of a broader approach used to assess the effect of emotional states on empathy, Trilla Gros et al. (2021) showed that an egocentric bias exists when perceiving ambiguous faces; if participants were happy, they were more likely to identify a facial expression as happy. This mood-congruency bias in emotion perception may be an important variable when designing empathy studies and calls for further examination.

#### Acute stress

Acute stress has been shown to modify information-processing steps, higher cognitive functions, and empathy (Hermans et al., 2014; Nitschke and Bartz, 2023; Shields et al., 2016). Stress, defined as a response occurring when external demands surpass one’s resources, triggers changes in affective, neural, cardiovascular, and hormonal processes (Lazarus and Folkman, 1984). A recent review by Nitschke and Bartz (2023) discusses the influence of acute stress on empathy. The authors report that evidence on enhanced empathy in the context of stress has been gathered in healthy samples and is debated under the term “tend-and-befriend.” The term describes increased prosocial activities due to stress that may also extend to empathic behavior (Taylor, 2006). Increased empathy in the aftermath of acute stress was also reported by Gonzalez-Liencres et al. (2016); participants undergoing the Trier Social Stress Test (TSST; Kirschbaum et al., 1993) evaluated pain experienced by a third person as more unpleasant compared to control participants without pre-experience of stress. In a functional magnetic resonance imaging (fMRI) study, Tomova et al. (2017), extended these findings by showing increased activity in brain structures associated with automatic empathy for others’ pain (e.g., the anterior insula, the anterior midcingulate cortex, the primary somatosensory cortex) after exposing male participants to a common fMRT stress-induction paradigm. It is important to note that both studies only targeted empathy for pain (Gonzalez-Liencres et al., 2016; Tomova et al., 2017). Future studies should address empathy for complex (positive and negative) emotions under stress as well.

As mentioned, acute stress is known to impair higher cognitive functions and adaptive behavior, compelling individuals to allocate their cognitive resources toward coping with the stressor (Hermans et al., 2014; Shields et al., 2016). This rationale leads to the expectation that acute stress reduces cognitive empathy (Nitschke and Bartz, 2023). Interestingly, empirical findings paint a mixed picture. Studies investigating simple emotion recognition as a key component of cognitive empathy endorse the beneficial effects of acute stress on cognitive empathy (Domes and Zimmer, 2019), though this may be restricted to positive emotions (von Dawans et al., 2020) or emotions expected to be more salient under stress such as disgust and surprise (Daudelin-Peltier et al., 2017). In contrast, authors such as Smeets et al. (2009), Wingenfeld et al. (2018), and Graumann et al. (2021) report null findings, while Wolf et al. (2015) found no effect of acute stress on cognitive empathy but a stress-induced enhancement of affective empathy. These inconsistencies between studies may arise due to the varying complexity and ecological validity of the different tasks used to assess emotion recognition (Nitschke and Bartz, 2023).

To add even more complexity, sex-specific effects have been reported, with female participants showing impaired or unaffected empathy under rising cortisol levels while male participants seemed to benefit from higher cortisol reactivity (Nitschke et al., 2022; Smeets et al., 2009). Because men generally exhibit a higher cortisol response to stress, direct comparisons are challenging (Nitschke and Bartz, 2023). Speculating on how cortisol might affect empathy on a mechanistic level, Nitschke and Bartz (2023) provide a framework for interpreting contradictory results. The authors suggest that cortisol may specifically target brain areas responsible for a meaningful self-other distinction. As such, enhanced empathy under stress may result from a failure to distinguish how far the perceived affect concerns one’s own emotional state, or the emotional state of another individual. The authors highlight a need for further research to identify additional mediators of the effects of acute stress on empathy beyond cortisol and other stress markers.

Taken together, the evidence shows that state empathy is context-dependent and amenable to various facilitative factors. These factors should be considered when devising empathy measurement tools and designing empathy intervention programs. This is crucial when tailoring interventions for specific groups such as individuals with autism spectrum disorder who may exhibit lower levels of trait empathy. Investigating the impact of these factors, whether individually or in combination, on improving the capacity to express state empathy is a promising avenue for future research and practice.

### Decreasing factors

Many of the aforementioned factors have the potential not only to heighten state empathy but also to diminish it. Consequently, in the following section, we summarize experimental conditions employed to reduce state empathy. Results provide valuable insights into the dynamics of state empathy and contribute to the development of more effective empathy measurement tools and interventions.

#### Focus of attention

Attention, or more precisely, the focus of attention, is one of the most prominent factors affecting state empathy. Gu and Han (2007) showed that attentional focus influences the activity of the neural network involved in empathy for pain. When participants focused on rating the painful experience of a person in a picture, the neural network related to empathy was active. If, however, participants were asked to count a specific aspect of the photos they saw (e.g., to count the number of identical hands), their attention shifted away from the emotional response, and the activity of the neural affective empathy network was decreased. Similarly, Fan and Han (2008) supported the assumption that attention has a moderating effect on the occurrence of empathy for pain. Specifically, redirecting cognitive resources away from someone else’s emotional signals can affect the initial perceptual aspect of empathy, leading to a diminished emotional reaction in the observer (Fan and Han, 2008). To the best of our knowledge, no study has adopted a similar approach to Gu and Han (2007) to investigate the influence of attentional focus on empathy in the context of more complex emotions.

#### Personal distress

Thus far, personal distress is one of the few emotional states investigated in terms of its direct influence on empathy (Kim and Han, 2018). Personal distress is defined as the tendency to experience negative feelings and discomfort when faced with the suffering of others. It is an emotional response that arises from empathy (Kim and Han, 2018) and results in the tendency to withdraw oneself from a stressor (Batson et al., 2009). As Preston et al. (2020) outline, impaired affective empathy may result from high levels of personal distress rather than a psychopathological deficit. Moreover, personal distress may occur if the self-regulation process after experiencing a shared affect is unsuccessful (Stevens and Taber, 2021). In this sense, experiencing personal distress leads to experiencing stress (Batson et al., 2009) and subsequent physical arousal. Deuter et al. (2018) recently investigated the influence of physiological arousal on affective empathy and found a negative relationship between arousal and self-reported affective empathy. They concluded that physiological arousal may diminish empathy (Deuter et al., 2018). Future research should investigate this connection to better understand the influence of physical arousal on empathy, particularly in experimental settings.

#### Blocking facial mimicry

It has been suggested that facial mimicry helps to better understand the perception of the emotional state of another person for basic and complex emotions (Drimalla et al., 2019). Blocking facial mimicry (e.g., by biting a pen or chewing gum) has been reported to decrease empathic processes (Stel and van Knippenberg, 2008). Several studies report that emotion recognition, an important component of empathy, is slower when facial mimicry is inhibited (Niedenthal, 2007; Stel and van Knippenberg, 2008) for a more comprehensive discussion see Hess and Fischer (2013). Thus, facial mimicry can be considered an embodied mediating factor and an example of situated emotional influence on state empathy.

#### Acute stress

Similar to other factors, acute stress may lead to a decrease, as well as an increase in state empathy. Because stress represents a state characterized by a reallocation of cognitive resources to stimuli other than the stressor, one might assume that available cognitive resources are predominantly needed to cope with the stressor in question. It is conceivable that under stress, available cognitive resources are invested in regulating one’s own emotional state rather than in showing empathy for the emotions of others. This is evident in Buruck et al. (2014) who found reduced empathy for pain in participants having undergone a TSST. However, this relation was moderated by participants’ emotion regulation capacities. Participants with stronger emotion regulation skills showed even higher deficits in empathic sharing. Initially, this may seem counterintuitive. One might assume that those skilled in emotion regulation require fewer cognitive resources for handling their own emotions, leaving resources available for empathizing with others. However, this does not guarantee a willingness to share others’ emotions under stress. Empathizing might amplify arousal and emotion regulation costs. Additionally, and as mentioned above, Smeets et al. (2009), Wingenfeld et al. (2018), and Graumann et al. (2021) report null findings, suggesting that acute stress may not consistently increase or decrease state empathy and that other factors play a role as well.

#### Characteristics of the target/stimulus material

Up to this point, we have discussed internal factors operating within the observer and thus shaping the expression of state empathy. Several external variables moderate the extent to which a person exhibits empathy in a given moment. For instance, factors such as the emotional valence of the stimulus (Drimalla et al., 2019; Gamsakhurdashvili et al., 2021a; Wolf et al., 2015), the observer’s relationship with the target including in-group and out-group biases (Cikara et al., 2014), and/or sex of the target (e.g., interaction with sex-hormone status of the female observer for cognitive empathy and affective empathy) may increase or decrease state empathy (Gamsakhurdashvili et al., 2021a; Gamsakhurdashvili et al., 2021b). Moreover, the ethnicity of the target (Xu et al., 2009) can modulate empathy depending on the valence of the context (Neumann et al., 2013). While a detailed discussion of these factors exceeds the scope of this review, they warrant consideration as potential covariates in future empirical studies.

It becomes apparent that while the distinction between increasing and decreasing factors is helpful for systemization, precise analyses are necessary to account for factors that can have both effects depending on nuanced individual and/or contextual differences. To account for this, all factors are summarized in Table 2.

**Table 2:** Overview of increasing and decreasing factors on state empathy.

Increasing factors
Acute stress (in the case of positive emotions, emotions expected to be more salient and mostly for men)
Attention (particularly in practicing mindfulness)
Compassion training
Emotion regulation strategy up-regulating intensity of one’s emotional state
Extrinsic motivation: social rewards
Intrinsic motivation: autonomy
Meditation
Mood congruency
Decreasing factors
Acute stress (however moderated by application of emotion regulation strategy and only reported for empathy for pain)
Attention (redirecting it away from the target; cognitive load)
Blocking of facial mimicry
Emotional valence of stimuli
In-group/Out-group bias
Personal distress
Physical arousal
Relationship proximity to the target

List in alphabetical order.

### Measuring trait and state empathy—what now?

Considering the complex nature of empathy and the multitude and versatility of factors influencing empathic responses described in the previous chapters, one can easily conclude that measuring empathy poses challenges. To mitigate them, this review developed simple and cost/time-efficient ways to enhance the validity of empathy measurements: (1) Measurement choice: as outlined in Chapter 2, studies investigating empathy frequently use empathy measurements without considering the inherent differences of the accessed concepts. The choice diagram depicted in Figure 1 provides a tool that can be used to decide what measurement should be used in a study to optimally quantify the specific aspect of empathy under investigation. (2) Time control: as outlined in Chapter 3, the time frame within which participants must indicate their empathic response can cause significant differences in results. To overcome this limitation, we recommend a simplified distinction between bottom-up and top-down processes in measuring empathy. To assess a bottom-up process, studies should integrate a forced response time for the empathic response, triggering a fast, intuitive reaction. To assess the general empathic response (consisting of bottom-up and top-down processes) studies should implement a time period during which participants cannot respond, ensuring that the top-down process has time to occur. (3) Confounding factors: as outlined in Chapter 4, the measurement of empathy is sensitive to several factors that can increase or decrease the empathic response. Since there is a risk that factors act in opposition to each other (meaning that factors that both increase and decrease empathic responses are present) causing null effects, it is important to control for the confounding effects at least to some degree. To facilitate the selection of appropriate study designs and measurements, Table 2 provides researchers with an overview of how different factors can influence empathy. We believe that the consideration of the three aforementioned elements (measurement choice, time control, and confounding factors) will enhance the validity and generalizability of empathy research results.

One interesting approach that more explicitly integrates biopsychological indicators would be to establish standardized test batteries that allow for the measurement of both cognitive and affective empathy through self-report (for trait empathy), performance-measures (for state empathy), and CNS-correlates (such as fMRI- or EEG-measures). One such battery is the so-called EmpaToM task (Kanske et al., 2015) for use in fMRI. The EmpaToM, which assesses cognitive and affective empathy and compassion, was shown to discriminate between the three corresponding types of neural pathways crucial for understanding others within the same functional-imaging task (initially developed in German; Kanske et al., 2015). Concretely, the neuronal correlates of affective empathy, ToM (theory of mind, i.e., cognitive empathy), and compassion are assessed while using dynamic video sequences of neutral and negative valence (related to suffering). The EmpaToM is now also available in English (Lantos et al., 2023). We would add that it is important to extend its scope by examining complex emotions of both positive *and* negative (and neutral) valence rather than focusing only on negative emotions (and neutral controls).

This review aimed to outline the benefits of differentiating between trait and state empathy on a theoretical and methodological level. In addition, we challenged current methodological approaches for measuring empathy. We elaborated on the theoretical aspects of trait and state empathy and discussed both increasing and decreasing factors of state empathy. Finally, we highlighted three factors that should be taken into consideration when designing future empathy studies.

We are aware that the current review comes with limitations. It is noteworthy that more aspects than those listed in this review shape empathy. For example, Weisz and Zaki (2017) summarize that expectations of the emphasizer due to their gender play a pivotal role, but only when the gender-related expectation is made conscious. In addition, and as stated above, other studies report that the valence of emotions interacts with the female’s menstrual cycle stage and therefore specifically affects empathy (Gamsakhurdashvili et al., 2021b; Wolf et al., 2015). While this paper’s selection of individual factors is literature-driven, it is not conceptualized as systematic due to the lack of previous approaches taken to address the topic. Despite these limitations, the review opens the possibility for future researchers to assess the distinction between trait and state empathy. Future research should aim to clarify inconsistencies in methodological approaches used to measure empathy.

## Conclusion

In conclusion, we believe that our approach is a valuable addition to the theoretical development of the construct. Empathy can be understood not only as a trait but a state. We call for researchers to consider that (both cognitive and affective) empathy is the result of both, bottom-up processes and top-down control mechanisms that are influenced by increasing and decreasing situational factors. Lastly, we highlight three efficient steps for improving existing trait and state empathy measures. Namely, researchers should choose the appropriate measurement tool, implement a time control during performance tasks, and control for confounding factors. Through this, we hope to increase the validity and generalizability of results in empathy research.
