# Supplementary material for: (State) empathy: how context matters
Source: Front Psychol. 2025 Feb 7;16:1525517. doi: 10.3389/fpsyg.2025.1525517 (PMC11878243; doi:10.3389/fpsyg.2025.1525517)
Supplement: Supplementary file 1 [file Table_1.docx]

**Appendix**

**Table A**

*List of sentences defining if the respective tool was categorized as trait or state measure*

| **Measurement Tool** | **Sentence for decision** |
| --- | --- |
| BLRI (Barrett-Lennard, 1962) | “The aspect of the empathic process that involves experiential recognition of perceptions or feelings that the other has directly symbolized and communicated may be termed empathic recognition. The aspect of sensing or inferring the implied or indirectly expressed content of the other's awareness is called empathic inference. In general these two aspects occur together in the empathic process, but their combination will vary from one relation- ship to another, and from moment to moment in a given relationship.” (Barrett-Lennard, 1962, p. 3) |
| EIS (Schutte et al., 1998) | “Emotional intelligence is generally conceptualized as a somewhat enduring, trait-like characteristic (e.g. Salovey and Mayer, 1990; Goleman, 1995; Mayer and Salovey, 1997).” (Schutte et al., 1998, p. 174) |
| ECRS (La Monica, 1981) | “Nineteen traits (as listed in Table 5) were studied in the multitrait-multimethod matrix. These were assessed by eight instruments using the following three methods of measurement: (a) self-reports, (b) a peer-rating scale, and (c) a client-rating scale. The eight instruments used in the matrix all measure traits that conceptually contribute to empathy. (La Monica, 1981, p. 390) |

| **Measurement Tool** | **Sentence for decision** |
| --- | --- |
| EQ (Baron-Cohen & Wheelwright, 2004) | “We expect that empathy traits are being assessed by the EQ, albeit in terms of the individual’s belief about their own empathic traits.” (Baron-Cohen & Wheelwright, 2004, p. 170) |
| JSPE (Hojat et al., 2001) | “These findings support the notion that empathy is a unique personal trait that is multidimensional (Davis, 1983).” (Hojat et al., 2001, p. 363) |
| HES (Hogan, 1969) | “Within this latter context, an empathic disposition can be regarded as the capacity to adopt a broad moral perspective, that is, to take "the moral point of view." (Hogan, 1969, p. 307) |
| MET-Core (Dziobek et al., 2011) | “The Multifaceted Empathy Test (MET; Dziobek et al., 2008) is an ecologically valid measure that allows for the separate assessment of the cognitive and emotional aspects of state empathy.” (Dziobek et al., 2011, p. 540) |

*Note*. All sentences are direct quotes taken out of the original manuscripts.

| PET (Lindeman et al., 2018) | “Although empathy is sometimes conceptualized as an ability, we follow here researchers who define empathy as a personality trait (Baron-Cohen & Wheelwright, 2004; Davis, 2006).” (Lindeman et al., 2018, p. 421) |
| --- | --- |
| QCAE (Reniers et al., 2011) | “Empathy is regarded as a favorable trait and participants might have been tempted to respond in a more socially desirable way.” (Reniers et al., 2011, p. 93) |
| SEE (Wang et al., 2003) | “Further, we conceptualized ethnocultural empathy as a trait that can be developed over time.” (Wang et al., 2003, p. 222) |
